# Delaying anti-VEGF therapy during the COVID-19 pandemic: long-term impact on visual outcomes in patients with neovascular age-related macular degeneration

**DOI:** 10.1186/s12886-023-02864-x

**Published:** 2023-04-17

**Authors:** Marco Nassisi, Francesco Pozzo Giuffrida, Paolo Milella, Simone Ganci, Andrea Aretti, Claudia Mainetti, Laura Dell’Arti, Chiara Mapelli, Francesco Viola

**Affiliations:** 1 Ophthalmological Unit, Fondazione IRCCS Ca’ Granda Ospedale Maggiore Policlinico, Milan, Italy; 2 Department of Clinical Sciences and Community Health, University of Milan, Via della Commenda 19, Milan, 20122 Italy

**Keywords:** Anti-VEGF, Neovascular age-related macular degeneration, Intravitreal injections, COVID-19

## Abstract

**Objectives:**

To evaluate the outcomes of delayed intravitreal injections (IVIs) caused by the outbreak of coronavirus disease 2019 (COVID-19), in patients with neovascular age-related macular degeneration (nAMD).

**Methods:**

nAMD patients with scheduled IVIs between March 1^st^ and April 30^th^, 2020 were stratified through a risk-based selection into a non-adherent group (NA-group) if they skipped at least one IVI and an adherent group (A-group) if they followed their treatment schedule. During the pandemic visit (v^0^), if a significant worsening of the disease was detected, a rescue therapy of three-monthly IVIs was performed. Multimodal imaging and best-corrected visual acuity (BCVA) findings were evaluated after 6 months (v^6^), compared between groups and with the visit prior the lockdown (v^−1^).

**Results:**

Two hundred fifteen patients (132 females, mean age: 81.89 ± 5.98 years) delayed their scheduled IVI while 83 (53 females, mean age: 77.92 ± 6.06 years) adhered to their protocol. For both groups, BCVA at v^0^ was significantly worse than v^−1^ (mean 4.15 ± 7.24 ETDRS letters reduction for the NA-group and 3 ± 7.96 for the A-group) but remained stable at v^6^. The two groups did not significantly differ in BCVA trends after 6 months and neither for development of atrophy nor fibrosis.

**Conclusions:**

A risk-based selection strategy and a rescue therapy may limit the long-term outcomes of an interruption of the treatment protocol in patients with nAMD.

## Introduction

After one year from its first outbreak and identification, the Coronavirus disease 2019 (COVID-19) has become pandemic, urging governments and global organizations to adopt several restrictive measures to limit the spread of the disease, protect high-risk categories of patients and avoid the over-charging of hospitals and intensive care units [1, 2].

Health-care providers adjusted to this evolving situation and first privileged urgent and emergency care. Then the continuity of treatment for chronic diseases was ensured. Finally, routine clinical practice could restart. All these steps had to be performed while protecting the safety of both patients and medical staff [3].

In the field of ophthalmology, one of the major issues was to deal with the great number of exudative neovascular age-related macular degeneration (nAMD) patients that require monthly intravitreal injections (IVIs) of anti-vascular endothelial growth factor (VEGF) agents [4, 5]. In Italy, the number of eyes that started an anti-VEGF therapy was about 2850 eyes/month between 2016 and 2019 [6]. After the spread of the European and the American outbreak, several medical societies worldwide elaborated recommendations for managing nAMD patients during the pandemic. Nevertheless, as Italy was the first Western country to experience the outbreak of the pandemic, there were no predefined guidelines, and several patients had their scheduled IVI treatment postponed or skipped as hospitals had to re-organize their activities and adapt their facilities to ensure the required standards of safety during the pandemic.

We recently reported that, during the first weeks of lockdown in March 2020, only one over three patients in our center adhered to the treatment protocol [7]. Most of the missed IVIs were promptly re-scheduled after a careful evaluation of the degree of urgency. Some studies showed the very short-term effects of the interruption of the regular treatment flow for these patients, mostly including a higher incidence of macular hemorrhages and a worse visual acuity (mean reported change could range between 0.05 and 1.09 LogMAR) [8–10]. However, as most of these patients re-started the treatment, the real clinical impact and the permanence of these changes is yet to be determined, as well as the factors that might influence it. In this study, we evaluated the visual impact of the discontinuity of the IVIs treatment in nAMD patients after 6-months of follow-up. This will serve clinicians to prepare strategies to mitigate vision loss in future scenarios of the COVID-19 pandemic evolution.

## Methods

### Patients and protocol

In this observational study, we included a total of 324 nAMD patients, who had at least one scheduled IVI between March 1^st^ and April 30^th^ 2020 at Fondazione IRCCS Ca' Granda, Ospedale Maggiore Policlinico, Milan, Italy. In our routine clinical practice, two protocols could be adopted: pro-re-nata (PRN) or observe-and-plan (O&P) [11, 12]. Specifically, O&P is adopted during the first three years of treatment of the macular neovascularization (MNV). Afterwards, once no disease activity is observed for at least 3 consecutive visits with IVI, a PRN protocol could be adopted with scheduled visits every 2–3 months. After September 2019, bevacizumab has become the only reimbursable drug for nAMD in Lombardy (i.e. where this study was conducted).

As no approved national or international guidelines were present before the end of March 2020, this cohort was divided into three priority groups (emergent, urgent and non-urgent), as previously described [7]. Briefly, “emergent” patients were the ones receiving the loading dose (first three anti-VEGF injections), or that had shorted the retreatment interval in the last three consecutive visits, or eyes with an active choroidal neovascularization at the last visit available (i.e. presence of evident exudation, increase of retinal fluid, hemorrhages on multimodal imaging) or patients with fellow eye BCVA ≤ 20/200 Snellen. “Urgent” patients had stable BCVA and disease activity in the last three consecutive visits. Finally, “nonurgent” patients had an IVI interval ≥ 3 months in the last three consecutive visits [7]. In general, while emergent patients were asked to follow their treatment schedule as set, IVIs for urgent and non-urgent patients were delayed (between 14 and 30 days) or even skipped. Of note, despite the priority assigned, some of the patients decided to postpone their treatment for personal reasons. In this study, all patients that delayed or skipped at least one injection during the recruitment time-frame were considered within the non-adherent group (NA-group) while all patients that followed a flawless protocol of treatment were considered within the adherent group (A-group) [13]. All patients were contacted by phone to discuss the different options. The study protocol was approved by the local institutional review board (Comitato Etico Milano Area 2) and adhered to the tenets of the Declaration of Helsinki.

For patients in the A-group, a clinical assessment was performed the day of the scheduled injection in the two-month recruitment time frame (v^0^) and six months later (v^6^).

For non-adherent patients, the same clinical assessment was done the day of the first performed injection (v^0^, regardless of the time frame) and six months after the first scheduled injection that was delayed or skipped between the 1^st^ of March and 30^th^ of April 2020 (v^6^). All patients in the NA-group were re-evaluated the day of the re-scheduled treatment through best corrected visual acuity (BCVA), fundus examination and spectral domain optical coherence tomography (SD-OCT).

In both groups, in case of evident worsening of the disease (loss of ≥ 2 ETDRS lines, newly formed macular hemorrhages, newly formed neovascular lesions, increasing of intra- or subretinal fluid on SD-OCT and/or appearance of subretinal hyperreflective exudation [SHE] compared to the last visit prior to the lockdown period [v^−1^]) three monthly intravitreal injections were scheduled for the patient before re-applying the previous protocol of treatment. In absence of this worsening, the treatment continued the O&P or PRN programs using the same time interval the patient reached before the pandemic. Furthermore, for all patients, a retrospective review of the charts was performed to collect data about the BCVA of v^−1^ and the number of injections in the previous 6 months before March 1^st^ 2020 (excluding patients in loading phase).

### Clinical and imaging assessment

An ophthalmic examination including BCVA with Early Treatment Diabetic Retinopathy Study (ETDRS) charts, slit-lamp examination and applanation tonometry was performed in each visit. Clinical assessment was then completed through fundus examination, short-wavelength fundus autofluorescence (SW-FAF) and SD-OCT (Heidelberg Spectralis HRA + OCT; Heidelberg Engineering, Dossenheim, Germany). Only one eye per patient was included in the study. In case both eyes were eligible, the eye with the worse BCVA was included.

For each patient, a cube of SD-OCT scans was obtained in high-speed mode and was used to measure the central retinal thickness (CRT). Dimension and density of the cube was determined by the previous exams of each patient as they were acquired in follow-up mode to allow a direct comparison throughout all patient’s visits (minimum number of B-scans: 49).

The type of MNV was assessed at v^−1^ according to angiographic (when available) and SD-OCT criteria [14–17]. At v^−1^ and v^6^, the SD-OCT cubes were graded for the presence and location of fluid, presence of complete retinal pigment epithelium and outer retinal atrophy (cRORA) and presence of subretinal hyper-reflective material (SHRM) [18, 19]. The latter was further identified as fibrosis or SHE through multimodal imaging including SD-OCT, infrared reflectance imaging and color fundus photographs when available [19]. SHE is an indicator of disease activity and, on SD-OCT, [19]. This qualitative assessment was performed by two graders (PM and FPG). In case of disagreement, the case was assessed through open adjudication. If no consensus was reached, the final decision was made by the medical director of the unit (FV).

### Statistical analysis

Statistical analysis was performed using SPSS Statistics V.20 (IBM Corp., Armonk, New York, USA). For categorical variables, X^2^ test was used for within and between groups comparisons. For continuous variables, first, the normal distribution was checked using the Shapiro–Wilk test; then, parametric and non-parametric tests were used accordingly. An analysis of covariance (ANCOVA) adjusted for age and sex was used to test the association between the two groups, protocol of treatment, MNV type and time between v^−1^ and v^0^ with the difference in BCVA between v^−1^ and v^6^. A *p*-value inferior to 0.05 was considered significant. All data are presented as mean ± standard deviation.

## Results

Between March 1^st^ and April 30^th^ 2020, 348 nAMD patients had at least one scheduled IVI. Twenty-four of them (0.7%) decided not to come back at the hospital until v^6^ and were excluded from the study. Among the remaining 324 (198 females [61.1%], age: 80.81 ± 6.25 years), 50 (15.4%) patients were in loading phase, 97 (29.9%) were treated using a PRN protocol, and 177 (54.6%) were in O&P. Most of the patients were treated using bevacizumab (309, 95.4%), while the remaining with ranibizumab (13, 4%) or aflibercept (2, 0.6%).

Only 88 subjects (27.2%, 55 females [62.5%], age: 77.92 ± 6.06 years) adhered to their treatment protocol; however, 5 of them (5.7%) did not come back for v^6^ and were excluded from the statistics. The remaining 236 subjects (72.8%, 143 females [60.6%], age: 81.89 ± 5.98 years) had delayed treatment. One hundred and twenty patients (50.8%) were rescheduled as they were non-emergent cases. One hundred and sixteen patients (49.2%), 29 of which were emergent (12.3%), ignored our recommendations because of the fear of COVID-19 infection (94, 39.8%), because of other cause of illness (e.g. other non-COVID-19-related infections or limited mobility due to domestic-related injuries; 12, 5.1%) or because of transportation-related difficulties (9, 3.8%). Financial status, which could also play a role in non-adherence, was not investigated. Twenty-one patients from the NA-group were excluded from the statistics as 4 (1.7%) were lost during the 6-month follow-up time, while 17 (7.2%) did not come back at v^6^.

### Non-adherent group

**Fig. 1:**
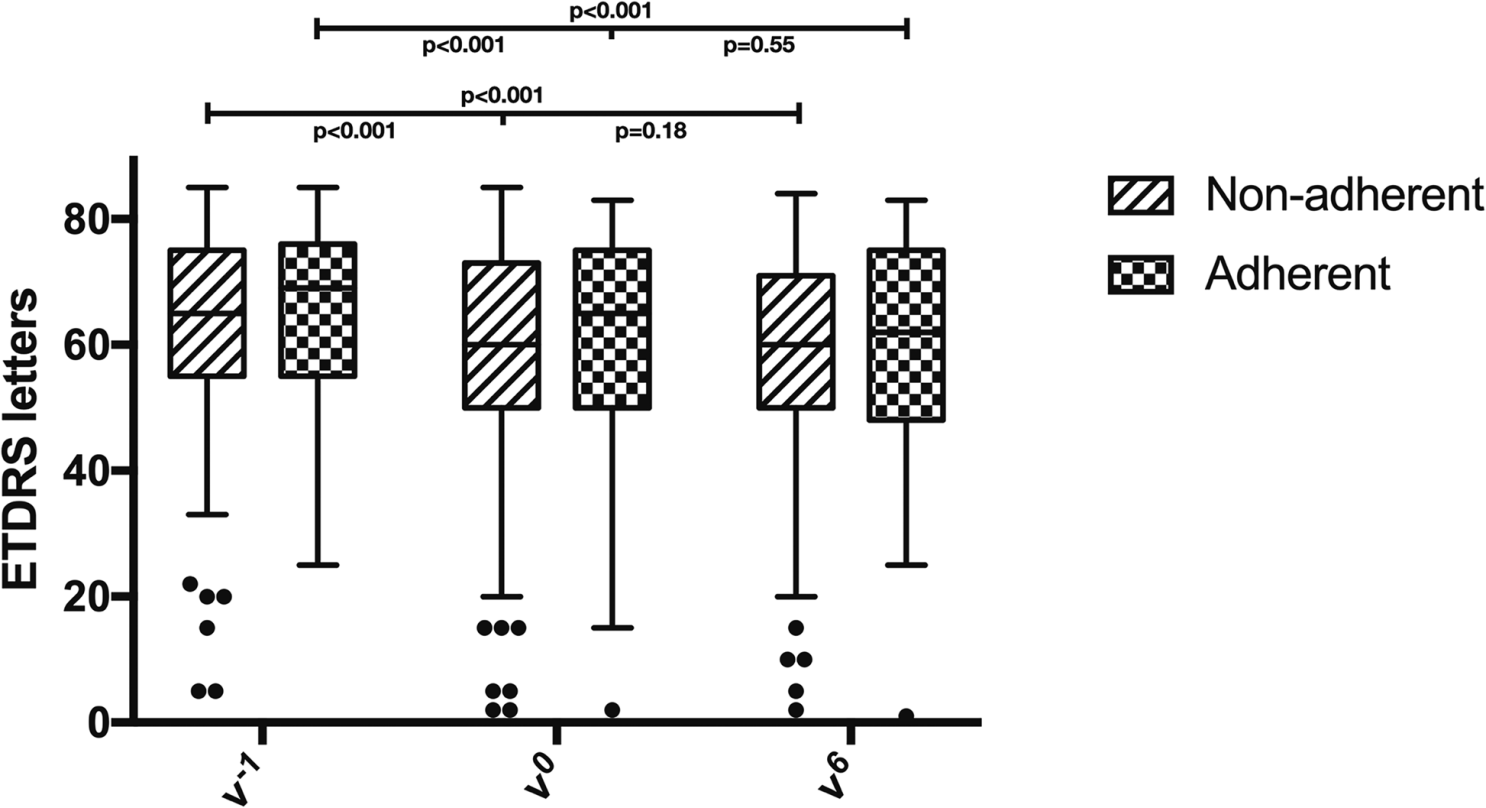
Box-and-whisker plot showing the best corrected visual acuity (in Early Diabetic Retinopathy Study [ETDRS] letters) of adherent and non-adherent groups at the three considered visits. All reported *p* values were calculated through paired t-test

Data from the NA-group are shown in Table 1. Most of the patients included in the NA-group group were treated with bevacizumab (203, 94.4%) and followed an O&P protocol (120, 55.8%). BCVA at v^−1^ was 62.72 ± 15.22 ETDRS letters which decreased significantly at v^0^ (58.57 ± 16.75 ETDRS letters, *p* < 0.001), while at v^6^ BCVA remained stable (57.87 ± 16.68 ETDRS letters; *p* = 0.18; Fig. 1). During the 6 months of the study these patients underwent an average of 2.69 ± 1.18 injections. The mean time between v^−1^ and v^0^ was 103.43 ± 32.15 days. At v^0^, 111 patients (47%) showed a significant worsening of the disease, therefore 3 monthly IVI were scheduled before re-applying their previous protocol of treatment.

**Table 1:** Demographic and clinical characteristics of the non-adherent and adherent groups (NA-group and A-group, respectively) with statistical comparisons

	NA-group (215 subjects)	A-group (83 subjects)	*P* value
Females: n (%)	132 (61.4)	53 (63.9)	0.70^†^
Age: mean (SD), years	81.89 (5.98)	77.92 (6.06)	< 0.001*
MNV type: n (%)			0.73^†^
1 (n:173)	129 (60)	44 (53)	
2 (n:46)	32 (14.9)	14 (16.9)	
3 (n:49)	33 (15.3)	16 (19.3)	
PCV (n:30)	21 (9.8)	9 (10.8)	
Treatment protocol: n (%)			0.001^†^
LP (n:39)	20 (9.3)	19 (22.9)	
PRN (n:90)	75 (34.9)	15 (18.1)	
O&P (n:169)	120 (55.8)	49 (59)	
Number of injections received in the 6 months before the study: mean (SD)	2.45 (1.24)	2.68 (1.16)	0.70*
**v**^**−1**^			
BCVA: mean (SD), ETDRS letters	62.72 (15.22)	64.43 (14.15)	0.38*
CRT: mean (SD), µm	346.63 (140.37)	347.66 (112.58)	0.98*
Fibrosis: n (%)			0.31^†^
Absent (n:191)	138 (64.2)	53 (63.9)	
Extrafoveal (n:28)	20 (9.3)	8 (9.6)	
Subfoveal (n:79)	57 (26.5)	22 (26.5)	
cRORA: n (%)			0.13^†^
Absent (n:167)	118 (54.9)	49 (59)	
Extrafoveal (n:53)	35 (16.3)	18 (21.7)	
Subfoveal (n:78)	62 (28.8)	16 (19.3)	
Type of fluid: n (%)			0.97^†^
Absent (n:39)	29 (13.5)	10 (12)	
Intraretinal Fluid (n:101)	75 (34.9)	26 (31.3)	
Subretinal Fluid (n:103)	76 (35.3)	27 (32.5)	
Both (n:55)	35 (16.3)	20 (24.1)	
Time between v^−1^ and v^0^: mean (SD), days	103.43 (32.15)	42.32 (20.79)	< 0.0001*
**v**^**0**^			
BCVA: mean (SD), ETDRS letters	58.57 (16.75)	61.43 (16.7)	0.19*
CRT: mean (SD)	363.56 (149.38)	340.35 (118.32)	0.21*
Δ BCVA v^−1^-v^0^	- 4.15 (7.24)	- 3.0 (7.96)	0.23*
Δ CRT v^−1^-v^0^	16.36 (114.93)	-7.31 (72.27)	0.082*
**v**^**6**^			
BCVA: mean (SD), ETDRS letters	57.87 (16.68)	60.81 (16.52)	0.17*
CRT: mean (SD), µm	351.57 (140.31)	346.4 (124.55)	0.77*
Fibrosis: n (%)			0.51^†^
Absent (n:177)	122 (56.7)	50 (60.2)	
Extrafoveal (n:23)	15 (7)	8 (9.6)	
Subfoveal (n:103)	78 (36.3)	25 (30.1)	
cRORA: n (%)			0.48^†^
Absent (n:146)	102 (47.4)	44 (53)	
Extrafoveal (n:57)	40 (18.6)	17 (20.5)	
Subfoveal (n:95)	73 (34)	22 (26.5)	
Type of fluid: n (%)			0.73^†^
Absent (n:65)	48 (22.3)	17 (20.5)	
Intraretinal Fluid (n:96)	66 (30.7)	30 (36.1)	
Subretinal Fluid (n:87)	66 (30.7)	21 (25.3)	
Both (n:50)	35 (16.3)	15 (18.1)	
Δ BCVA v^−1^-v^6^	- 4.85 (7.71)	- 3.63 (8.74)	0.24*
Δ CRT v^−1^-v^6^	4.37 (96.69)	-1.27 (86.62)	0.64*
Number of injections received in the 6 months of the study: mean (SD)	2.69 (1.18)	3.58 (1.35)	< 0.0001*

*SD* Standard deviation, *MNV* Macular neovascularization, *PCV* Polypoidal choroidal vasculopathy, *LP* Loading phase, *PRN* Pro-re-nata, *O&P* Observe-and-plan, *BCVA* Best corrected visual acuity, *CRT* Central retinal thickness, *cRORA* complete retinal pigment epithelium and outer retinal atrophy

^*^ Unpaired t-test

^†^ Pearson’s X^2^ test

At the SD-OCT assessment, CRT initially increased (346.63 ± 140.37 µm at v^−1^ and 363.56 ± 149.38 µm at v^0^, *p* = 0.038), while at v^6^ remained stable compared to v^0^ (351.57 ± 140.31 µm; *p* = 0.063). Intra- and/or subretinal fluid were present in the 86.5% of patients at v^−1^; at v^0^ this percentage remained stable (86.9%), while it slightly but significantly decreased at v^6^ (77.7%, *p* = 0.049).

A sub-analysis conducted according to the type of MNV and treatment protocol (Table 2) showed that, compared to v^−1^, BCVA was significantly lower at v^6^ for type 1 (from 64.51 ± 13.94 to 60.13 ± 15.62 ETDRS letters, *p* < 0.001), type 2 (from 55.58 ± 16.14 to 51.18 ± 17.83 ETDRS letters, *p* = 0.006) and type 3 (from 64.21 ± 13.71 to 57.48 ± 14.96 ETDRS letters, *p* < 0.001) lesions and for patients in PRN protocol (from 64.52 ± 14.44 to 59.6 ± 15.59 ETDRS letters, *p* < 0.001) and O&P protocol (from 63.39 ± 13.81 to 58.56 ± 16 ETDRS letters, *p* < 0.001; Table 2). The same sub-analysis for CRT showed a significant decrease for patients in loading phase (from 435.57 ± 253.51 µm to 346.74 ± 161.7 µm, *p* = 0.01) and in O&P protocol (from 326.6 ± 93.87 µm to 346.26 ± 114.520 µm, *p* = 0.008).

**Table 2:** Analysis of functional and imaging outcomes in the non-adherent group (NA-group), according to macular neovascularization (MNV) type and treatment protocol

**NA-group** **n:215**		**BCVA (ETDRS letters, mean (SD))**			**CRT (µm, mean (SD))**			**Subretinal fibrosis (n)**			**cRORA (n)**			**Type of fluid (n)**		
		**v**^**−1**^	**v**^**6**^	***p***	**v**^**−1**^	**v**^**6**^	***p***	**v**^**−1**^	**v**^**6**^	***p***	**v**^**−1**^	**v**^**6**^	***p***	**v**^**−1**^	**v**^**6**^	***p***
**MNV Type**	**1** **n:129**	64.51 (13.94)	60.13 (15.62)	< 0.0001*	331.73 (86.01)	339.97 (101.11)	0.14*	No: 78 Yes: 51	No: 66 Yes: 63	0.084^†^	No: 75 Yes: 54	No: 67 Yes: 62	0.19^†^	No: 16 IRF: 42 SRF: 49 Both: 22	No: 29 IRF: 42 SRF: 41 Both: 17	0.035^†^
	**2** **n:32**	55.58 (16.14)	51.18 (17.83)	0.006*	377.82 (159.16)	376.64 (171.93)	0.92*	No: 17 Yes: 15	No: 14 Yes: 18	0.309^†^	No: 18 Yes: 14	No: 17 Yes: 15	0.50^†^	No: 5 IRF: 9 SRF: 11 Both: 7	No: 7 IRF: 8 SRF: 12 Both: 5	0.81^†^
	**3** **n:33**	64.21 (13.71)	57.48 (14.96)	< 0.0001*	301.82 (97.28)	300.18 (93.9)	0.94*	No: 26 Yes: 7	No: 25 Yes: 8	0.50^†^	No: 10 Yes: 23	No: 6 Yes: 27	0.195^†^	No: 5 IRF: 21 SRF: 5 Both: 2	No: 9 IRF: 13 SRF: 5 Both: 6	0.17^†^
	**PCV** **n:21**	60.70 (20.02)	56.96 (20.29)	0.097*	450.35 (290.22)	443.61 (250.67)	0.88*	No: 17 Yes: 4	No: 17 Yes: 4	0.652^†^	No: 15 Yes: 6	No: 12 Yes: 9	0.26^†^	No: 3 IRF: 3 SRF: 11 Both: 4	No: 3 IRF: 3 SRF: 8 Both: 7	0.53^†^
**Treatment Protocol**	**LP** **n:20**	53.3 (21.18)	50.30 (21.03)	0.25*	435.57 (253.51)	346.74 (161.7)	0.01*	No: 15 Yes: 5	No: 13 Yes: 7	0.366^†^	No: 7 Yes: 13	No: 5 Yes: 15	0.366^†^	No: 3 IRF: 4 SRF: 5 Both: 8	No: 8 IRF: 6 SRF: 4 Both: 2	0.023^†^
	**PRN** **n:75**	64.52 (14.44)	59.6 (15.59)	< 0.0001*	351.67 (146.67)	358.16 (168.54)	0.50*	No: 43 Yes: 32	No: 39 Yes: 36	0.311^†^	No: 40 Yes: 35	No: 34 Yes: 41	0.207^†^	No: 15 IRF: 22 SRF: 28 Both: 10	No: 2 IRF: 19 SRF: 23 Both: 12	0.71^†^
	**O&P** **n:120**	63.39 (13.81)	58.56 (16.0)	< 0.0001*	326.6 (93.87)	346.26 (114.520)	0.008*	No: 80 Yes: 40	No: 70 Yes: 50	0.115^†^	No: 71 Yes: 49	No: 63 Yes: 57	0.181^†^	No: 11 IRF: 49 SRF: 43 Both: 17	No: 19 IRF: 41 SRF: 39 Both: 21	0.33^†^

*SD* Standard deviation, *MNV* Macular neovascularization, *PCV* Polypoidal choroidal vasculopathy, *LP* Loading phase, *PRN* Pro-re-nata, *O&P* Observe-and-plan, *BCVA* Best corrected visual acuity, *CRT* Central retinal thickness, *cRORA* complete retinal pigment epithelium and outer retinal atrophy, *IRF* Intra-retinal fluid, *SRF* Sub-retinal fluid

^*^ Unpaired t-test

^†^ Pearson’s X^2^ test

### Adherent group

Data from the A-group are shown in Table 1. Most of the patients included in the A-group group were treated with bevacizumab (80, 96.4%) and followed a O&P protocol (49, 59%). BCVA at v^−1^ was 64.43 ± 14.15 ETDRS letters which decreased significantly at v^0^ to 61.43 ± 16.7 ETDRS letters and then remained stable to 60.81 ± 16.52 ETDRS letters at v^6^ (both *p* < 0.001 compared with v^−1^, but *p* = 0.55 between v^0^ and v^6^, Fig. 1). During the 6 months of the study these patients underwent an average of 3.58 ± 1.35 injections. At v^0^, 43 patients (51.8%) showed a significant worsening of the disease, therefore 3 monthly IVI were scheduled before re-applying their previous protocol of treatment. The mean time between v^−1^ and v^0^ was 42.32 ± 20.79 days. At the SD-OCT assessment, CRT at v^−1^ was 347.66 ± 112.58 µm and remained stable at v^0^ (340.35 ± 118.32 µm, *p* = 0.89) and v^6^ (346.4 ± 124.55 µm, *p* = 0.42). Intra- and/or subretinal fluid were present in the 88% of patients at v^−1^; at v^0^ this percentage slightly decreased to 80.7%, while remaining constant at v^6^ (79.5%).

A sub-analysis conducted according to the type of MNV and treatment protocol (Table 3) showed that, compared to v^−1^, BCVA was significantly lower at v^6^ for type 1 (from 66.68 ± 13.19 to 63.25 ± 15.19 ETDRS letters, *p* = 0.019) and 3 (from 59.06 ± 17.31 to 53.44 ± 20.70 ETDRS letters, *p* = 0.032) lesions and for patients in O&P protocol (from 63.88 ± 15.47 to 59.73 ± 17.39 ETDRS letters, *p* = 0.001; Table 1). The same sub-analysis for CRT showed a significant decrease only for patients in loading phase (from 410.79 ± 98.11 µm to 361.79 ± 90.87 µm, *p* = 0.036).

**Table 3:** Analysis of functional and imaging outcomes in the adherent group (A-group), according to macular neovascularization (MNV) type and treatment protocol

**A-group** **n:83**		**BCVA (ETDRS letters, mean (SD))**			**CRT (µm, mean (SD))**			**Subretinal fibrosis (n)**			**cRORA (n)**			**Type of fluid (n)**		
		**v**^**−1**^	**v**^**6**^	***p***	**v**^**−1**^	**v**^**6**^	***p***	**v**^**−1**^	**v**^**6**^	***p***	**v**^**−1**^	**v**^**6**^	***p***	**v**^**−1**^	**v**^**6**^	***p***
**MNV Type**	**1** **n:44**	66.68 (13.19)	63.25 (15.19)	0.019*	363.89 (127.63)	354.5 (125.57)	0.49*	No: 30 Yes: 14	No: 28 Yes: 16	0.41^†^	No: 31 Yes: 13	No: 29 Yes: 15	0.41^†^	No: 5 IRF: 11 SRF: 19 Both: 9	No: 9 IRF: 14 SRF: 13 Both: 8	0.42^†^
	**2** **n:14**	64.14 (13.84)	60.36 (13.88)	0.071^§^	333.14 (83.0)	315.07 (65.6)	0.17^§^	No: 8 Yes: 6	No: 8 Yes: 6	0.648^†^	No: 10 Yes: 4	No: 7 Yes: 7	0.22^†^	No: 1 IRF: 4 SRF: 2 Both: 7	No: 4 IRF: 4 SRF: 3 Both: 3	0.23^†^
	**3** **n:16**	59.06 (17.31)	53.44 (20.70)	0.032^§^	323.81 (95.64)	371.81 (170.37)	0.077^§^	No: 8 Yes: 8	No: 6 Yes: 10	0.361^†^	No: 2 Yes: 14	No: 2 Yes: 14	0.70^†^	No: 3 IRF: 8 SRF: 1 Both: 4	No: 2 IRF: 9 SRF: 1 Both: 4	0.95^†^
	**PCV** **n:9**	63.44 (16.26)	62.67 (17.14)	0.74^§^	333.33 (104.08)	310.33 (89.47)	0.33^§^	No: 7 Yes: 2	No: 7 Yes:2	0.50^†^	No: 6 Yes: 3	No: 6 Yes: 3	0.69^†^	No: 1 IRF: 3 SRF: 5 Both: 0	No: 2 IRF: 3 SRF: 4 Both: 0	0.71^†^
**Treatment Protocol**	**LP** **n:19**	65.58 (13.26)	64.16 (14.14)	0.50^§^	410.79 (98.11)	361.79 (90.87)	0.036^§^	No: 14 Yes: 5	No: 10 Yes: 9	0.157^†^	No: 14 Yes: 5	No: 10 Yes: 9	0.157^†^	No: 1 IRF: 5 SRF: 6 Both: 7	No: 2 IRF: 8 SRF: 4 Both: 5	0.51^†^
	**PRN** **n:15**	64.8 (13.59)	60.07 (16.86)	0.066^§^	289.53 (76.77)	293.13 (93.56)	0.88^§^	No: 9 Yes: 6	No: 9 Yes:6	0.50^†^	No: 7 Yes: 8	No: 8 Yes: 7	0.50^†^	No: 4 IRF: 5 SRF: 4 Both: 2	No: 5 IRF: 5 SRF: 4 Both: 1	0.78^†^
	**O&P** **n:49**	63.88 (15.47)	59.73 (17.39)	0.001*	340.98 (116.81)	356.73 (140.57)	0.16*	No: 30 Yes: 19	No: 30 Yes: 19	0.582^†^	No: 28 Yes: 21	No: 26 Yes: 23	0.42^†^	No: 5 IRF: 16 SRF: 17 Both: 11	No: 10 IRF: 17 SRF: 13 Both: 9	0.49^†^

*SD* Standard deviation, *MNV* Macular neovascularization, *PCV* Polypoidal choroidal vasculopathy, *LP* Loading phase, *PRN* Pro-re-nata, *O&P* Observe-and-plan, *BCVA* Best corrected visual acuity, *CRT* Central retinal thickness, *cRORA* complete retinal pigment epithelium and outer retinal atrophy, *IRF* Intra-retinal fluid, *SRF* Sub-retinal fluid;

^*^ Unpaired t-test

^†^ Pearson’s X^2^ test

^§^Wilcoxon matched pair test

### Comparison between groups

Direct comparison between NA- and A-groups at v^−1^ showed no significant differences for demographic characteristics, number of injections in the previous 6 months, BCVA, CRT and SD-OCT characteristics (Table 1). Only the distribution of the treatment protocols among the 2 groups was statistically different, with a higher percentage of patients in loading phase in the A-group. Indeed, this was related to the initial selection of patients, which was not randomized and was directed to the inclusion of the urgent cases (e.g. patients in loading phase) into the A-group.

Despite a small number of patients in both groups developed fibrosis and/or cRORA from v^−1^ to v^6^, this increase was not significant.

No differences emerged in v^0^ and v^6^ except for the number of injections received in the 6 months of the study, which was higher for the A-group (*p* < 0.001). The ANCOVA did not reveal any significant association between any considered independent variables and the difference of BCVA between v^−1^ and v^6^.

## Discussion

This study aimed to assess the long-term impact of the delayed care on the visual and anatomic outcomes of patients with nAMD and the outcomes of the strategies adopted to face the COVID-19 pandemic.

In real-life studies that specifically analyzed the rate of discontinuation of therapy in nAMD patients, the percentage of patients who did not comply to their treatment protocol could vary between 2.9% and 53%, depending on the considered time frame [20–23]. At the beginning of the COVID-19 emergency, only about 25% of nAMD patients complied to their scheduled treatment at our center. Regular IVI retreatment is important to retain the vision in patients with macular diseases and skipping injections can have a negative effect [22, 24]. *Chong Teo KY *et al*.* in *The RAMPS Study*, proved that in treatment-naive nAMD patients commencing anti-VEGF monotherapy, a delayed re-treatment (defined as two or more skipped IVIs while the disease is active), causes a significant decrease in the VA gain at the end of the first year of treatment. Furthermore, a longer delay was associated with poorer vision outcomes [22].

In our cohort, the NA-group had an average delay of treatment of 60 days. This was comparable to the time delay registered by *Borrelli *et al*.* who reported the short-term effects of the pandemic on the visual acuity of patients with nAMD in another referral ophthalmological center in Milan [10]. The short-term visual outcomes were also similar, however, no long-term outcomes were reported, and a control group was absent. Interestingly, in our study, both groups showed a reduction of BCVA which was already evident at v^0^ and sustained through v^6^, despite there was no significant increase in the incidence of fibrosis and/or cRORA,. In the NA-group, IVI skipping was considered the main reason for the BCVA decrease. Indeed, the inclusion of some emergent (i.e. more severe) patients in the NA-group, who chose to postpone the treatment for personal reasons, may have influenced the outcome. In the A-group, we hypothesize that the BCVA decrease could be related to different reasons: first, 51.8% (43 patients) of the adherent eyes showed a significant worsening of the disease at v^0^, which was comparable to the 51.6% (111 patients) of the NA-group; given the recent switch to bevacizumab for the majority of patients, treatment was still being tailored on most of them, hence it is likely that all patients were being under-treated before the beginning of the study (only 13.5% and 12% of patients had a dry retina according to CATT criteria at v^−1^ in the NA- and A-group, respectively [25]). Second, the allocation of the patients in the two groups was not random and only the emergent and clinically worse cases were treated regularly within the lockdown; third, a delay inferior to 15 days was considered acceptable to be part of the A-group in this study but it is impossible to exclude that even a short delay of treatment, above all in these cases, might have determined a reduction of the BCVA.

At the end of the study, when full activity was regained, both groups maintained their visual acuity stable. It is possible that the initial risk-based selection of patients limited the worsening of their condition; furthermore, patients with important exudation at v^0^ were directly treated with three monthly IVI despite their previous protocol of treatment. Other strategies have been proposed to reduce the number of visits, in favor of a continuous treatment: *Sacconi *et al*.* proposed the “Triple and Plan” regimen which consists in evaluating the patient every three IVIs [26]; *Korobelnik *et al*.* suggest to reduce the treatment interval to the last effective treatment interval and use this for fixed dosing [27]. All these strategies were likely inspired by the observe-and-plan regimen which first privileged the number of injections to the number of assessing visit, to improve the compliance [12]. Indeed, these strategies may ensure less exposure, more safety and higher compliance. The latter was one of the most important issue during the study and especially at the beginning of the pandemic; we already reported that the adherence rate among “emergent” patients during the lockdown period was only 0.60 [7]; as an example, 51.3% of the patients in loading phase (20 subjects) refused to receive treatment at first, despite our recommendations and reassurances [7]. At the same time, the group of 24 patients (0.7% of the total) who were not visited nor treated until six months after the beginning of the lockdown (non-persistent group as for the definition by Okada et al. [13]), had an average decrease of 6.29 ± 13.89 ETDRS letters in BCVA. All these patients were on their 2nd or 3rd year of treatment and with a relatively stable condition. Most of them relied on the self-assessment of their visual acuity at home and decided to return at the clinic only when they subjectively perceived a significant worsening of the disease or when they judged the environmental situation safe enough.

In conclusion, the results of this study demonstrate the importance of the continuity of therapy in patients with nAMD, while showing that a careful risk-based selection and a rescue therapy with three consecutive monthly IVIs may be effective in limiting the long-term outcomes of an interruption of the treatment protocol. The study also helped us in advising patients during the second wave of COVID-19, giving them the means to decide whether to pursuing treatment while weighting the risk of the pandemic.

## Data Availability

The datasets used and/or analysed during the current study are available from the corresponding author on reasonable request.
